# Predictive Features Specific to FIGO IIIA1 Ovarian Cancer: What Drives Prognosis

**DOI:** 10.1245/s10434-026-19895-5

**Published:** 2026-06-02

**Authors:** Matteo Bruno, Davide Arrigo, Marco Paratore, Giustina Lopopolo, Valerio Gallotta, Andrea Rosati, Marco D’Indinosante, Anna Fagotti

**Affiliations:** 1 Dipartimento Scienze della Salute della Donna, del Bambino e di Sanità Pubblica, Fondazione Policlinico Universitario Agostino Gemelli, IRCCS, Rome, Italy; 2 Dipartimento Scienze della Vita e Sanità Pubblica, Università Cattolica del Sacro Cuore, Rome, Italy

**Keywords:** Epithelial ovarian cancer, Host inflammatory markers, Lymph node ratio, Lymphadenectomy, Nodal metastasis, Para‑aortic lymph nodes, Prognostic factors

## Abstract

**Background:**

FIGO stage IIIA1 ovarian cancer, defined by lymph node–only metastasis, represents a biologically distinct pattern of spread within epithelial ovarian cancer. Patients with IIIA1 disease experience more favorable outcomes than those with peritoneal dissemination; however, the prognostic determinants within this subgroup remain incompletely defined and underexplored.

**Methods:**

A narrative review was conducted by using concept‑based searches. Eligible reports included those specifically addressing stage IIIA1 or providing extractable node‑positive subsets within stage III cohorts. Given heterogeneity in surgical eras, lymphadenectomy practices, and histologic composition, no quantitative pooling was performed.

**Results:**

Nodal size and the IIIA1(i)/(ii) subdivision did not consistently correlate with survival. In contrast, nodal topography showed signals of prognostic relevance in selected cohorts. The lymph node ratio (LNR) emerged as the most robust determinant of outcome. Systematic pelvic and para‑aortic lymphadenectomy, typically involving retrieval of ≥10–20 nodes, was linked to improved staging accuracy and long‑term survival. Tumor microenvironment features provided additional prognostic stratification, while IIIA1‑specific molecular data remained limited.

**Conclusions:**

In FIGO IIIA1 ovarian cancer, nodal topography and burden represent the most reliable prognostic indicators. Systematic lymphadenectomy appears beneficial in appropriately selected patients, and tumor microenvironment features offer complementary risk stratification. Future studies should focus on genomically characterized IIIA1 cohorts to refine risk‑adapted surgical and maintenance strategies.

**Supplementary Information:**

The online version contains supplementary material available at 10.1245/s10434-026-19895-5.

In epithelial ovarian cancer (EOC), the presence and pattern of metastatic dissemination at diagnosis remain the strongest determinants of prognosis. The 2014 revision of the International Federation of Gynecology and Obstetrics (FIGO) system, clarified in 2021, formally recognized this heterogeneity by defining stage IIIA1 as disease confined to lymph-node metastases and by introducing subclasses IIIA1(i) and IIIA1(ii) for micro- and macrometastatic deposits, respectively. FIGO gave formal expression to a view shared among gynecologic oncologists: node-positive, peritoneum-negative ovarian cancer often behaves as a biologically distinct entity, with different survival outcomes.^1–3^

In clinical practice, prognostic assessment frequently centers on nodal size; however, emerging data indicate that nodal topography (pelvic vs. para-aortic; infrarenal vs. suprarenal) and nodal burden-quantified by the lymph node ratio (LNR; positive nodes divided by nodes removed) may be more discriminative than size thresholds alone.^4–10^ However, two independent cohorts did not demonstrate reproducible survival differences between IIIA1(i) and IIIA1(ii), challenging a purely size-based stratification within IIIA1 and shifting attention toward metrics that better reflect extent of nodal disease.^11,12^

Across datasets, higher LNR thresholds (e.g., >0.25 or ≥0.50) correlate with stepwise worsening of survival, supporting the inclusion of LNR in routine pathology reporting.^4,5^ These observations open the discussion on surgical strategy in apparent early and IIIA1 disease. Registry analyses focused on IIIA1 populations suggest that systematic pelvic and para-aortic lymphadenectomy (often operationalized as the removal of ≥10–20 nodes) is associated with improved long-term survival.^7,8,13^

Histotype remains foundational-high-grade serous ovarian carcinoma (HGSOC) predominates in IIIA1-yet histology alone does not explain the observed outcome variability.^14^ Finally, contemporary systemic therapy has evolved substantially. For many patients, a central question is how to integrate first-line maintenance strategies into a risk-adapted plan for node-only disease, given the lack of IIIA1-specific randomized evidence and the generally more favorable baseline prognosis of this subgroup.^7,8,14^

The purpose of this review is to synthesize current evidence on prognostic and predictive factors in FIGO IIIA1 EOC, focusing on nodal distribution and burden, the role of systematic lymphadenectomy, and the contribution of histology. Our goal is to provide a comprehensive overview of prognostic factors in ovarian cancer with nodal-only spread, delineate the key evidence gaps that must be addressed to move from retrospective associations to evidence-based standards, and support further development of a risk-stratification framework.^5–8,11–17^

## Methods

### Study Design

This article is a narrative review informed by a structured, concept-based literature search. The purpose was to summarize prognostic (and clinically relevant predictive) determinants in FIGO stage IIIA1 epithelial ovarian cancer (e.g., nodal burden by lymph node ratio, para-aortic distribution, residual disease status), focusing on outcomes of progression-free/disease-free survival (PFS/DFS) and overall survival (OS). Given the expected heterogeneity across staging eras, surgical practice, and reporting standards, evidence was synthesized narratively and no quantitative pooling was planned. No formal protocol registration was performed given the narrative scope.

### Search Strategy

Searches were conducted in PubMed/MEDLINE, Embase, and Web of Science from database inception to December 31, 2025, without language restrictions. Search terms combined concepts for epithelial ovarian/fallopian tube/primary peritoneal cancer with FIGO IIIA1/node-only disease and prognostic metrics. To enhance transparency, the full PubMed strategy is reported (Appendix S1). We considered original clinical studies evaluating prognosis in FIGO stage IIIA1, or reporting mixed stage III cohorts with an extractable node-positive/IIIA1 subset. In addition, selected secondary sources (e.g., staging updates, pathology reporting standards, and focused reviews) were retained to contextualize interpretation where IIIA1-specific primary data were limited. Nonepithelial histologies, imaging-only studies without survival data, and editorials were excluded; publications were also excluded when outcomes were not relevant (or time-to-event endpoints were not reported) or when data were insufficient.

### Study Selection and Data Extraction

After deduplication, records were screened for relevance and full texts were assessed for eligibility. For included publications, we extracted study design, population, staging definition, nodal variables (burden, topography, LNR, and lymphadenectomy extent), treatment context, follow-up, and reported effect estimates (hazard ratios [HR] with 95% confidence interval [CI] when available). Findings were organized narratively by prespecified domains. A total of 965 records were identified through database searches, PubMed (*n* = 290), Web of Science (*n* = 345), and Embase (*n* = 330). After removal of duplicates, 580 records remained and were screened for relevance. Of these, 460 were excluded based on title and abstract screening. The remaining 120 full-text articles were assessed for eligibility. Seventy-six articles were excluded for the following reasons: inability to extract a node-positive subgroup or mixed-stage reports without separable lymph node positive assessment (*n* = 43), nonrelevant outcomes/insufficient time-to-event reporting (*n* = 21), conference abstracts, overlapping datasets or incomplete reports without sufficient data (*n *= 11), and missing data despite author contact (n = 1). A total of 44 publications were included.

## Results

Table 1 summarizes the main published studies investigating prognostic factors in node-positive epithelial ovarian cancer available in literature.

**Table 1:** Summary of main studies evaluating prognostic determinants in FIGO stage IIIA1 epithelial ovarian cancer

Study	Design	Overall population (no. patients)	Primary endpoint	Key prognostic factors
Suh et al., 2013	Retrospective (multicenter)	870	OS, PFS	Nodal distribution, Residual disease
Ataseven et al., 2014	Retrospective (multicenter)	809	OS	Lymph Node Ratio, Residual disease
Gasimli et al., 2016	Retrospective (multicenter)	218	PFS, OS	Nodal distribution, Substage (IIIA1i/ii), Residual disease
Kleppe et al., 2016	Retrospective (multicenter)	3658	RS	Lymphadenectomy extent, Chemotherapy
Pereira et al., 2016	Retrospective (multicenter)	116	OS, PFS	Substage (IIIA1i/ii), Nodal distribution, Lymphadenectomy extent
Zhou et al., 2016	Retrospective (registry-based)	5926	OS, CSS	Lymph node ratio, Lymphadenectomy extent
Ayhan et al., 2018	Retrospective (multicenter)	229	OS	Lymph node ratio, Nodal distribution
Şahin et al., 2018	Retrospective (multicenter)	179	DFS, OS	Disease distribution (Nodal vs. Peritoneal), Grade
Wang et al., 2018	Retrospective (registry-based)	4069	OS, CSS	Lymph node ratio, Lymphadenectomy extent
Nie et al., 2019	Retrospective (single center)	265	PFS, OS	Lymph node ratio
Tong et al., 2019	Retrospective (registry-based)	7815	OS	Lymph Node Ratio, Lymphadenectomy extent
Aslan et al., 2020	Retrospective (single center)	62	PFS, OS	Lymph node ratio
Chang et al., 2020	Retrospective (single center)	192	PFS, OS	Lymphadenectomy extent, Chemotherapy
Bizzarri et al., 2021	Retrospective (multicenter)	639	DFS, OS	Lymphadenectomy extent, Stage
Matsuoka et al., 2022	Retrospective (single center)	895	PFS, OS	Substage (IIIA1i/ii), Lymph Node Ratio, Nodal distribution
Bizzarri et al., 2023	Retrospective (single center)	359	DFS, OS	Inflammatory markers (NLR, SII), Stage
Yang et al., 2023	Retrospective (single center)	134	PFS, OS	Disease distribution (Nodal vs. Peritoneal)
Laven et al., 2025	Retrospective (registry-based)	5407	RS	Lymphadenectomy extent, Chemotherapy

Study design, population, primary endpoints, and key prognostic factors analyzed across main retrospective and registry-based cohorts

OS, overall survival; PFS, progression-free survival; DFS, disease-free survival; CSS, cancer-specific survival; RS, relative survival; NLR, neutrophil-to-lymphocyte ratio; SII, systemic immune-inflammation index

### IIIA1(i) versus IIIA1(ii): Micrometastasis versus Macrometastasis

In the Mayo restaged cohort, the largest nodal deposit >10 mm was not independently associated with outcomes (univariate risk relative [RR] 2.57–3.00 vs. microscopic nodes; not significant), and subclassification into IIIA1(i) (≤10 mm) versus IIIA1(ii) (>10 mm) did not separate survival on multivariable analysis.^2^ Subsequent single-center validation likewise showed numerically worse PFS/OS for IIIA1(ii) but no statistical significance (5-year PFS 68.7% vs. 58.1%, *p* = 0.58; 5-year OS 83.1% vs. 80.2%, *p* = 0.44).^11^ Gasimli et al. observed a numerically lower 3-year PFS in IIIA1(ii) compared with IIIA1(i) (62.6% vs. 90%), but the adjusted hazard ratio for PFS 2.3 (95% CI 0.45–11.58) was not significant.^12^ Collectively these findings suggest that micro- vs. macro- classification does not independently associate with outcome once broader nodal and surgical variables are considered.

### Nodal Distribution

In nonserous stage III EOC, Şahin et al. demonstrated substantially better outcomes for nodal-only spread versus peritoneal involvement: 5-year DFS 66.4% vs. 37.6% (*p* = 0.002) and 5-year OS 74.4% vs. 54% (*p* = 0.011).^16^ Although not restricted to IIIA1, this stage III analysis supports the general principle that lymphotropic dissemination behaves more favorably than peritoneotropic routes.

IIIA1-restricted comparisons of pelvic versus para-aortic metastases are scarce, and most prospective or registry datasets that informed the 2014/2021 FIGO revisions did not report outcomes within IIIA1 by nodal topography. Consequently, no high-level evidence shows that pelvic versus infra-renal para-aortic involvement alone independently stratifies survival among node-only patients once nodal burden and tumor biology are accounted for.

However, some studies report that involvement of only para-aortic lymph nodes is associated with a more favorable prognosis compared to pelvic lymph node involvement or simultaneous involvement of both pelvic and para-aortic lymph nodes.^18^ In type II (high-grade serous) ovarian cancer, para-aortic lymph node metastasis confers better survival than pelvic node involvement, while in type I (low-grade/endometrioid), pelvic node involvement may be less unfavorable.^11,12,18^

Nevertheless, the International Collaboration on Cancer Reporting (ICCR), including the College of American Pathologists and the European Society of Pathology, recognizes these anatomical distinctions in the FIGO staging system but does not currently recommend further stratification of stage IIIA1 by nodal location in clinical guidelines.^19^

### Nodal Burden: The Lymph Node Ratio

Among nodal metrics, the lymph node ratio (LNR)—the proportion of positive nodes among those removed—emerges as a robust predictor across studies. Indeed, in a multicenter stage III HGSOC cohort, LNR ≥0.50 vs. <0.10 conferred HR 2.7 (95% CI 1.42–5.18; *p* < 0.001) for death.^5^ Similarly, in another large EOC cohort, LNR >0.25 vs. ≤0.25 associated with worse OS (HR 1.44, 95% CI 1.04–2.00; *p* = 0.028).^4^ The LNR appears to be a consistent prognostic marker in node-positive ovarian cancer, including IIIA1-enriched cohorts. Multiple retrospective studies and large database analyses demonstrate that a higher LNR is associated with significantly decreased overall and progression-free survival in node-positive ovarian cancer, including those with isolated nodal involvement (stage IIIA1).^4,20–23^ This prognostic impact is observed across histologic subtypes and remains significant regardless of the absolute number of positive nodes or the extent of lymphadenectomy, with proposed cutoffs (e.g., LNR >0.09, >0.25, >0.42) consistently linked to poorer outcomes.^4,21,23^ Current staging systems, such as the 2014 FIGO classification, recognize the favorable prognosis of patients with isolated nodal involvement but do not yet incorporate LNR into risk stratification or treatment guidelines, despite evidence supporting its prognostic value. Interpretation of LNR is influenced by the total number of nodes retrieved, which varies widely across institutions and surgical approaches.^24^ Low node counts (<10) may produce unstable denominators and artificially inflate LNR. Moreover, published cutoffs (0.09, 0.25, 0.50) differ owing to heterogeneity in surgical quality, histologic composition, and statistical modeling.^24^

### Extent of Lymphadenectomy

Some studies on the size of metastatic lymph nodes in FIGO stage IIIA1 ovarian cancer demonstrate a trend toward worse outcome, with smaller and fewer nodal metastases (IIIA1(i), ≤10 mm) generally associated with better progression-free and overall survival than larger or more numerous nodes (IIIA1(ii), >10 mm).^2,11,19^ However, differences in survival between IIIA1(i) and IIIA1(ii) are not always statistically significant. In a dedicated validation study of the 2014 FIGO subclassification, 5-year PFS was 68.7% in IIIA1(i) versus 58.1% in IIIA1(ii) (*p* = 0.58), and 5-year OS 83.1% versus 80.2% (*p* = 0.44).^11^ These data support reporting the largest nodal metastasis in millimeters but caution against over-interpreting IIIA1(i)/(ii) as a stand-alone discriminator in small series.^2,11,19^ In IIIA1, lymphadenectomy serves both diagnostic and potentially therapeutic roles, and retrospective data suggest improved staging accuracy and survival in node-positive populations.^25^

Evidence favors systematic lymphadenectomy (LND)—often defined as removal of ≥10–20 nodes—in early and IIIA1 cohorts.^7,8,13^ In a large multicenter study of apparent early-stage EOC (the population that contains most IIIA1 cases), comprehensive pelvic plus para-aortic LND was independently associated with improved DFS (5-year DFS 79.7% with comprehensive LND vs. 68.3% with no LND; HR 0.52, 95% CI 0.37–0.73; *p* < 0.001), suggesting both staging and possible therapeutic effects.^13^ National registry work from the Netherlands, similarly reports that removing ≥10 nodes was linked with higher 10-year relative survival in clinical early-stage EOC after adjustment, reinforcing a threshold effect consistent with surgical quality metrics.^8^ Earlier nationwide data also debated that an “adequate” LND (≥10–20 nodes) should be standard for accurate staging in early-stage EOC.^7^ Table 2 summarizes the role of nodal prognostic factors evaluated across studies.

**Table 2:** Summary of nodal prognostic factors evaluated across studies including FIGO stage IIIA1 epithelial ovarian cancer

Study population	Nodal factor	Effect size	Statistical significance
46 FIGO stage IIIA1 (Matsuoka et al., 2022)	IIIA1(i) vs. IIIA1(ii) LNR (<0.045 vs. ≥0.045) Nodal distribution (PLN or PAN vs. PLN+PAN)	IIIA1(i) vs. IIIA1(ii): 5-year PFS: 68,7% vs. 58.1% 5-year OS: 83,1% vs. 80.2% LNR (<0.045 vs. ≥0.045) 5-year PFS: 55.9% vs. 65.1% 5-year OS: 89.7% vs. 76.5% Nodal distribution (PLN or PAN vs. PLN+PAN): 5-year PFS: 53.1% vs. 77.8% 5-year OS: 85.2% vs. 81.4%	IIIA1(i) vs. IIIA1(ii): n.s. LNR: n.s. Nodal distribution: n.s.
218 FIGO stage IIIA-IIIC (28 FIGO stage IIIA1) (Gasimli et al., 2016)	IIIA1(i) vs. IIIA1(ii)	3-year PFS: 90% vs. 62.6% (HR 2.3, 95% CI 0.45–11.58)	n.s.
116 FIGO stage IIIA-IVB (100% N+; 23 FIGO stage IIIA1) (Pereira et al., 2016)	IIIA1(i) vs. IIIA1(ii) LND extent (1–10, 11–20, 21–30, 31–40, >40)	IIIA1(i) vs. IIIA1(ii): OS: RR 0.74 (CI 95% 0.42–1.30) PFS: RR 0.64 (CI 95% 0.29–1.42)	IIIA1(i) vs. IIIA1(ii): n.s. LND extent: n.s
229 FIGO stage IIIA-IIIC (100% N+; 31 FIGO stage IIIA1) (Ayhan et al., 2017)	LNR (≥0.50; 0.10-0.49; <0.10)	LNR 0.10-0.49 vs. <0.10: OS: HR 2.3 (95% CI 1,460–3,803) LNR ≥0.50 vs. <0.10: OS: HR 2.7 (95% CI 1.42–5.19)	LNR 0.10-0.49 vs. <0.10: *p* = 0.003^b^ LNR ≥0.50 vs. <0.10: *p* < 0.001^b^
809 FIGO stage I-IV (398 N+ FIGO stage III-IV) (Ataseven et al., 2014)	LNR (>0.25 vs. ≤0.25)	OS: HR 1.44 (95% CI 1.04–2.00)	*p* = 0.028
639 FIGO stage I-IIIA1 (58 FIGO stage IIIA1) (Bizzarri et al., 2020)	LND extent (≥20; 1-19; 0)	LND extent (≥20 and 1-19) vs. 0: 5-year DFS: 79.7% vs. 68.3%; HR 0.52 (95% CI 0.37–0.73) LND extent ≥20 vs. 1-19: 5-year DFS: 79.7% vs. 76.5%	LND extent (≥20 or 1-19) vs. 0: *p* < 0.001 LND extent ≥20 vs. 1-19: n.s.
5407 FIGO stage IA-IIA or IIIA1 (209 FIGO stage IIIA1) (Laven et al., 2025)	LND extent (≥10; 1-9; 0)	LND extent (≥10 and 1-9) vs. 0: 10-year RS: 83.3% vs. 73.3%; 10-year RER 0.56 (95% CI 0.48–0.66)	*p* < 0.001
62 FIGO stage IIIA-IIIC (100% N+) (Aslan et al. 2020)	LNR (≤0.09 vs. >0.09)	LNR >0.09: HR 3.51 (95% CI 1.54–8.03) (PFS) LNR (≤0.09 vs. >0.09): 5-year PFS: 61.7% vs. 32% 5-year OS: 72.8% vs. 54.7%	LNR >0.09: *p* = 0.003 (PFS) LNR >0.09 vs. ≤0.09: *p* = 0.046 (OS); *p* = 0.043 (PFS)
5926 FIGO stage IIIC (100% N+) (Zhou et al. 2016)	LNR (≤0.42 vs. >0.42) LND extent (1-10; 11-20; >20)	LNR >0.42: CSS: HR 1,896 (95% CI 1,709–2,104) OS: HR 1,679 (95% CI 1,454–1,939) LNR (≤0.42 vs. >0.42): 5-year CSS: 53.1% vs. 34.7% 5-year OS: 50.4% vs. 32% LND extent: CSS: HR 0.998; OS: HR 0.996	LNR >0.42: *p* < 0.001 (CSS); *p* < 0.001(OS) LNR >0.42 vs. ≤0.42: *p* < 0.001 (CSS);* p* < 0.001 (OS) LND extent: n.s.
265^c^ FIGO stage III (100% N+; 42 FIGO stage IIIA1) (Nie et al. 2019)	LNR (>0.25 vs. ≤0.25)	PFS: HR 1.96 (95% CI 1.44–2.68) OS: HR 2.8 (95% CI 1.97-3.96)	*p* < 0.001 (PFS) *p* = 0.001 (OS)
10,878 FIGO stage I-IV (4069 FIGO stage III) (Wang et al., 2018)	LND extent (1; 2–3; 4–5; 6–15; 16–30; ≥31) LNR (<0.1; 0.1-0.4; 0.4-1)	LND extent: OS: HR 0.949 (0.917–0.982) CSS: HR 0.947 (0.915–0.981) LNR: OS: HR 1.234 (1.163–1.310) CSS: HR 1.221 (1.148–1.298)	LND extent: *p* = 0.002 (OS) *p* = 0.003 (CSS) LNR: *p* < 0.001 (OS) *p* < 0.001 (CSS)
7815 FIGO stage III-IV (100% N+) (Tong et al. 2019)	LNR (≥0.42 vs. <0.42) LND extent (>10 nodes)	LNR: OS: HR 1.526 (95% CI 1.415-1.647) LND extent: OS: 11–20 LN: HR 0.816 (95% CI 0.76–0.876); OS: >20 LN: HR 0.745 (95% CI 0.692–0.801)	LNR: *p* < 0.0001 LND extent: *p* < 0.0001

Study populations, effect sizes, and statistical significance are reported for key nodal metrics for multivariate analyses and FIGO stage IIIA1 (when available)

*n.s.* not significant; *LNR* lymph node ratio; *LND* lymphadenectomy; *PLN* pelvic lymph nodes; *PAN* paraaortic lymph nodes; *PFS* progression-free survival; *OS* overall survival; *CSS* cancer-specific survival; *RR* relative risk; *HR* hazard ratio; *CI* confidence interval; *RS* relative survival; *RER* relative excess risk; *N+* positive lymph node(s)

^a^“Retroperitoneal-only” group, including 35 patients with extra-pelvic peritoneal disease <2 cm

^b^Data reported on overall population

^c^Overall number of patients (178 “training cohort”, 87 “validation cohort”); data reported for overall validation cohort

### Histology

Available evidence indicates that histotype remains a foundational classifier in EOC, but IIIA1-specific, histology-stratified outcome data are limited. HGSOC predominates among node-only cases, and broader stage III cohorts consistently show that patterns of lymphotropic spread carry prognostic weight independent of size-based nodal metrics.^12,26,27^ Among the subtypes, endometrioid and low-grade serous carcinomas are associated with the most favorable survival outcomes, while HGSOC has intermediate prognosis, and clear cell, mucinous, and carcinosarcoma subtypes are linked to poorer survival in advanced stages, including IIIA1.^26–31^ Specifically, population-based and SEER registry studies demonstrate that patients with endometrioid and low-grade serous histology have higher 5-year survival rates compared with those with high-grade serous, clear cell, or mucinous carcinomas at stage III.^28–30^ Mucinous and clear cell carcinomas are independent predictors of poor prognosis, with shorter time to progression and death compared with serous histology, even after controlling for age and residual disease.^26,31^

### Tumor Microenvironment

Prognosis across EOC is broadly shaped by the interplay between the tumor microenvironment and systemic immune–inflammatory status, with tumor-infiltrating lymphocytes (TILs) and circulating inflammation indices emerging as complementary biomarkers. High Systemic Immune-Inflammation Index (SII, platelets × neutrophils / lymphocytes) is consistently associated with poorer survival in ovarian cancer overall and specifically in FIGO stage III–IV subgroups (OS HR 2.67; DFS HR 2.22).^32^ In an “apparent early-stage” cohort (including FIGO stages I–II plus IIIA1), both SII ≥1000 and an elevated neutrophil-to-lymphocyte ratio (NLR; neutrophils/lymphocytes) ≥3 were associated with worse 3-year disease-free survival (SII: 71.4% vs. 85.9%; NLR: 74.6% vs. 85.8%), and SII ≥1000 also with worse 3-year overall survival (94.6% vs. 98.2%), although stage remained the dominant driver after adjustment.^17^

## Discussion

When analyzing prognostic determinants within FIGO stage IIIA1 EOC, while nodal size has traditionally attracted interest, the available data suggest that nodal topography (i.e., “where” metastases are located) could have greater prognostic weight than simple size thresholds (“how big”). Preoperative imaging able to distinguish pelvic versus para‑aortic involvement, infrarenal versus suprarenal para‑aortic extension should inform both the extent of surgical planning and postoperative risk stratification. In contrast, while size cutoffs (e.g., >10 mm short‑axis) retain value for diagnostic staging, they have not consistently shown independent prognostic significance once node‐only disease is established.^2,11,12^ Emerging evidence suggests that extra-abdominal node-only ovarian cancer may represent a biologically distinct phenotype.^33^ Proposed mechanisms include reduced mesothelial adhesion and differential expression of lymphangiogenic factors (e.g., VEGF-C/D), potentially favoring lymphatic dissemination over peritoneal spread. Although these data mainly derive from studies on supradiaphragmatic lymph nodes, they support the hypothesis that also FIGO stage IIIA1 disease may likewise reflect a specific interaction between tumor biology and the lymphatic microenvironment.^33^

Among nodal metrics, LNR emerges as the most relevant predictor within the IIIA1 subset. Higher LNR thresholds (for example >0.25 or ≥0.50) are associated with incremental decrements in survival.^4,5,20–23^ Because LNR encapsulates both tumor burden (numerator) and surgical/staging thoroughness (denominator), it reduces the instability inherent in simple counts of positive nodes. Together, LNR plus nodal topography explain a part of the observed heterogeneity in the IIIA1 subgroup.

Regarding the subdivision of IIIA1 into micro‑ versus macro‑metastatic nodal disease (IIIA1(i) versus IIIA1(ii)) and the relevance of nodal size thresholds, two separate analyses failed to demonstrate reproducible survival separation between micrometastatic and macrometastatic nodal disease. While intuitively larger nodal deposits might imply worse prognosis, this binary split did not hold once more informative variables (e.g., LNR, distribution) were considered. Similarly, nodal size thresholds (for example >10 mm) have not proven independently prognostic after multivariate adjustment.^2,11,12,19^

The extent and quality of lymphadenectomy act both as a prognostic marker of outcome. Evidence from early‑stage and IIIA1‑enriched populations indicates that systematic pelvic and para‑aortic dissection (often defined as retrieval of ≥10–20 nodes, extending at least to the renal veins) associates with improved long‑term outcomes and more accurate staging.^7,8,13^ In IIIA1 specifically, such benefits are plausible; improved staging accuracy and removal of occult micrometastases may both contribute.

Prognosis in EOC is largely determined by the interaction between the tumor microenvironment and systemic immune–inflammatory status, with TILs and circulating inflammation indices providing complementary prognostic information; however, their effects are histotype- and treatment-dependent, and robust evidence at the level of individual FIGO substages remains scarce. Also, host‑inflammatory indices (including NLR and SII) might offer complementary, low‑cost stratification in apparent early and IIIA1 cohorts.^17,32^ Higher intraepithelial CD8+ TIL density is associated with improved survival. However, stromal immunosuppressive programs, compartment-specific immune topography, and chemotherapy–driven microenvironment remodeling can change the direction of these associations, supporting integrated immune–stromal risk stratification over single biomarkers.^17,32,34–38^ These parameters associate with recurrence and reflect the interplay of tumor biology and host response. Nonetheless, most cohorts do not report nodal-only spread subgroups, leaving the applicability and effect size of immune-related prognostic effects in stage IIIA1 uncertain. This limitation is particularly relevant because stage IIIA1 outcomes may be more strongly driven by lymph-node and host-immune interactions than peritoneal tumor burden. Accordingly, substage-specific analyses and prospective validation represent key next steps to identify the minority of stage IIIA1 tumors who behave aggressively despite “limited” anatomic spread.

Histology and molecular markers remain foundational, but in the IIIA1 context they are currently under‑resolved. Histotype (high‑grade serous versus non‑serous) remains central to risk stratification, and although specific IIIA1‑subgroup data are sparse, molecular features (BRCA/HRD status, TP53/CCNE1 alterations) likely retain prognostic or predictive relevance.^29–31^

Finally, does first‑line maintenance therapy (for example with anti‐angiogenic agents or PARP inhibitors) carry equal weight in IIIA1? Unfortunately, first‑line maintenance trials did not report IIIA1‑specific subgroup data; extrapolation from mixed stage III–IV populations suggests that relative benefits may persist, but absolute gains could be smaller in IIIA1 because baseline risk is lower.^39–43^

**Fig. 1:**
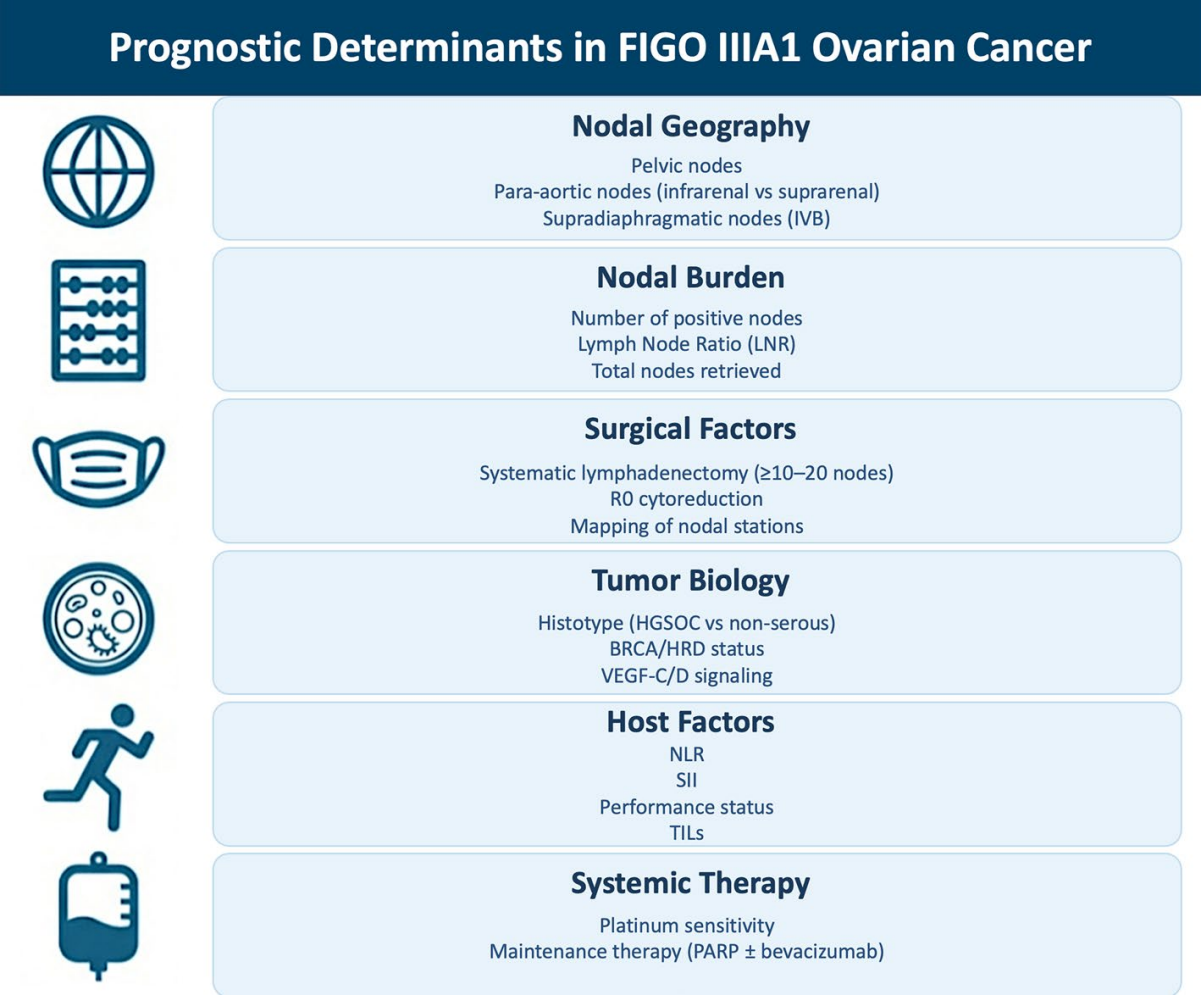
Framework summarizing the major prognostic determinants in FIGO stage IIIA1 epithelial ovarian cancer. The diagram illustrates six key domains: nodal topography, nodal burden, surgical factors, tumor biology, host inflammatory markers, and systemic therapy. *LNR* lymph node ratio; *HGSOC* high-grade serous ovarian carcinoma; *BRCA* breast cancer gene; *HRD* homologous recombination deficiency; *VEGF* vascular endothelial growth factor; *NLR* neutrophil-to-lymphocyte ratio; *SII* systemic immune-inflammation index; *TILs* tumor-infiltrating lymphocytes

In the absence of dedicated randomized data for IIIA1, a rational approach is to integrate LNR, nodal topography, histology/HRD/BRCA status into a shared decision‑making process that balances toxicity and expected benefit (Fig. 1).

### Strengths and Limitations of the Evidence

Although the available evidence consistently suggests a prognostic role for nodal topography, LNR, and lymphadenectomy extent in FIGO stage IIIA1 ovarian cancer, its overall reliability remains limited by several methodological constraints. The main strength is the consistency of prognostic signals across independent datasets; however, most studies are retrospective and extend across heterogeneous staging eras, with variable lymphadenectomy practices and potential selection bias favoring healthier patients treated in specialized centers.^41–43^ Restaging according to the 2014/2021 FIGO criteria is often performed retrospectively, introducing misclassification risk. In addition, many studies enrolled mixed stage III populations, with only small or indirectly extractable IIIA1 subsets; therefore, although these data provide useful signals, their applicability to pure node-only FIGO IIIA1 disease should be interpreted cautiously. Adjustment for key confounders, including histology, BRCA/HRD status, residual disease, and maintenance treatment, is inconsistent, and IIIA1-specific molecular data remain sparse. Reporting of discrimination metrics and external validation of prognostic models is also limited. Therefore, current findings should be considered clinically informative but not definitive when interpreting the prognostic role of nodal topography, LNR, and lymphadenectomy extent. These limitations underscore the need for standardized reporting frameworks and dedicated prospective IIIA1-specific cohorts to validate whether prognostic factors identified in broader stage III populations retain the same magnitude and clinical relevance in this subgroup.

### Future Directions

Future research should prioritize prospective, genomically annotated IIIA1 cohorts to validate the prognostic relevance of nodal topography and LNR and to determine whether these metrics warrant incorporation into future FIGO revisions. Beyond nodal count-based metrics, the histopathologic pattern of nodal involvement may further refine risk within node-only disease. Extracapsular extension represents a different biological phenotype, plausibly reflecting loss of containment, stromal invasion and enhanced metastatic competence. In primary node-positive ovarian cancer, extracapsular growth has been described and explored as a prognostic correlate.^44,45^ More recently, in the setting of isolated lymph nodal recurrence, a recent study from our group showed that extracapsular extension, particularly macroscopic extension (>2 mm), is common and independently associated with worse post-relapse outcomes.^46^ Although the ECEROC study focuses on recurrence rather than de novo IIIA1, it strengthens the conceptual framework that “nodal burden” should not be reduced to size alone and that future IIIA1-specific models should incorporate nodal architecture (intra- or extracapsular extension) in addition to size, topography and LNR.

Emerging multi-omics approaches such as single-cell–informed T-cell–related transcriptomic signatures may enable finer prognostic stratification in ovarian cancer by capturing the tumor immune microenvironment, but current evidence is largely retrospective and in silico, requiring prospective validation in contemporary treatment cohorts.^47^

Future prognostic stratification may be improved by integrating molecular pathology biomarkers (e.g., RB1 loss and its interaction with BRCA status/p53 patterns) with clinical staging, as large multicenter datasets show these features provide stage-adjusted, histotype-specific survival information that could refine risk groups beyond FIGO alone.^48^

Collectively, substage-specific analyses within FIGO staging are essential to refine risk stratification and personalize prognostic assessment. Also, dedicated subgroup analyses within randomized trials are needed to clarify the absolute benefit of maintenance therapies in node-only disease.

## Conclusions

In FIGO IIIA1 ovarian cancer, prognosis appears to be driven more by nodal topography and nodal burden than by the IIIA1(i)/(ii) subdivision alone. The LNR and nodal distribution may refine risk stratification, while systematic lymphadenectomy remains relevant for accurate staging and may improve outcomes in selected patients. However, current evidence remains largely retrospective and heterogeneous, and prospective, genomically annotated IIIA1 cohorts are needed to validate integrated prognostic models and clarify the absolute benefit of maintenance strategies in node-only disease.

## Supplementary Information

Below is the link to the electronic supplementary material.

Supplementary file1 (DOCX 14 KB)

## Data Availability

No new data were created or analyzed in this study. Data sharing is not applicable to this article.
