# Supplementary material for: Predictive Features Specific to FIGO IIIA1 Ovarian Cancer: What Drives Prognosis
Source: Ann Surg Oncol. 2026 Jun 2;33(9):8380–9. doi: 10.1245/s10434-026-19895-5 (PMC13452780; doi:10.1245/s10434-026-19895-5)
Supplement: Supplementary file 1 [file 10434_2026_19895_MOESM1_ESM.docx]

**Appendix S1 – Search strategy**

( "ovarian cancer" OR "epithelial ovarian cancer" OR "ovarian carcinoma" OR "ovarian neoplasm" OR "fallopian tube cancer" OR "fallopian tube carcinoma" OR "primary peritoneal cancer" OR "primary peritoneal carcinoma" )

AND

( “FIGO III” OR "FIGO IIIA1" OR "FIGO stage IIIA1" OR "stage IIIA1" OR IIIA1 OR "IIIA1(i)" OR "IIIA1(ii)" OR "node-only" OR "node only" OR "nodal-only" OR "nodal only" OR

("isolated" AND ("lymph node" OR nodal) AND (metastasis OR metastases)) OR

("retroperitoneal" AND (metastasis OR metastases)) )

AND

( "lymph node ratio" OR "nodal burden" OR "positive nodes" OR

lymphadenectomy OR lymphadenectomies OR "lymph node dissection" OR

"nodal metastasis" OR "lymph node metastasis" OR

pelvic OR "para-aortic" OR paraaortic OR infrarenal OR suprarenal )

AND

( prognos* OR survival OR "overall survival" OR "progression-free survival" OR

"disease-free survival" OR "hazard ratio" )
